# Effect of COVID-19 pandemic on utilisation of community-based mental health care in North-East of Italy: A psychiatric case register study

**DOI:** 10.1017/S2045796023000100

**Published:** 2023-04-11

**Authors:** E. Prina, F. Tedeschi, D. Salazzari, T. Botte, M. Ballarin, L. Rabbi, G. Imperadore, S. Roccato, S. Nicolaou, M. Ruggeri, F. Gomez, A. Lasalvia, F. Amaddeo

**Affiliations:** 1 Department of Neurosciences, Biomedicine and Movement Science, Section of Psychiatry, University of Verona, Verona, Italy; 2 Mental Health Department, Local Health District n. 9 of Verona, Verona, Italy

**Keywords:** Community mental health, epidemiology, health service research, psychiatric services

## Abstract

**Aims:**

WHO declared that mental health care should be considered one essential health service to be maintained during the coronavirus disease 2019 (COVID-19) pandemic. This study aims to describe the effect of lockdown and restrictions due to the COVID-19 pandemic in Italy on mental health services’ utilisation, by considering psychiatric diagnoses and type of mental health contacts.

**Methods:**

The study was conducted in the Verona catchment area, located in the Veneto region (northeastern Italy). For each patient, mental health contacts were grouped into: (1) *outpatient care*, (2) *social and supportive interventions*, (3) *rehabilitation interventions*, (4) *multi-professional assessments*, (5) *day care*. A ‘difference in differences’ approach was used: difference in the number of contacts between 2019 and 2020 on the weeks of lockdown and intermediate restrictions was compared with the same difference in weeks of no or reduced restrictions, and such difference was interpreted as the effect of restrictions. Both a global regression on all contacts and separate regressions for each type of service were performed and Incidence Rate Ratios (IRRs) were calculated.

**Results:**

In 2020, a significant reduction in the number of patients who had mental health contacts was found, both overall and for most of the patients’ characteristics considered (except for people aged 18–24 years for foreign-born population and for those with a diagnosis of schizophrenia. Moreover, in 2020 mental health contacts had a reduction of 57 096 (−33.9%) with respect to 2019; such difference remained significant across the various type of contacts considered, with rehabilitation interventions and day care showing the greatest reduction. Negative Binomial regressions displayed a statistically significant effect of lockdown, but not of intermediate restrictions, in terms of reduction in the number of contacts. The lockdown period was responsible of a 32.7% reduction (IRR 0.673; *p*-value <0.001) in the overall number of contacts. All type of mental health contacts showed a reduction ascribable to the lockdown, except social and supportive interventions.

**Conclusions:**

Despite the access to community mental health care during the pandemic was overall reduced, the mental health system in the Verona catchment area was able to maintain support for more vulnerable and severely ill patients, by providing continuity of care and day-by-day support through social and supportive interventions.

## Introduction

Since the first documented cases of coronavirus disease 2019 (COVID-19) on 21st of February 2020, Italy has been one of the most affected countries after China (Istituto Superiore di Sanità, 2020). The rapid spread of SARS-CoV-2 throughout the Italian territory, the dangerousness of the disease, required a great number of resources not promptly available at the beginning (Lasalvia *et al*., 2021). On 9 March, the government imposed a national quarantine, called ‘lockdown’, to reduce the spread of the coronavirus, limiting the movement of the population except for reasons of necessity (e.g., work, health visits, etc.). On 3 May, the Italian Government put an end to the lockdown period (Governo Italiano, 2020), gradually resuming economic activities, and slowly allowing for movements within the Region with a different level of restrictions based on the trend of pandemic emergency (Hale *et al*., 2021).

In this framework, high-stress levels were observed on (1) the general population, with an increase of the level of depression and anxiety (Fiorillo *et al*., 2020; Moccia *et al*., 2020); on (2) people with mental conditions, with a higher vulnerability in terms of physical and mental health (de Girolamo *et al*., 2020; Monteleone *et al*., 2021); on (3) healthcare workers, with a more frequent burn-out and psychological problems (emotional exhaustion 58.3%; low professional efficacy 57.5%; and high cynicism 39.2%) (Lasalvia *et al*., 2022); and on (4) general practitioners (GPs) (44.7% reported COVID-19-related traumatic events, 36% reported symptoms of anxiety, 17.9% symptoms of at least moderate depression and 25.4% symptoms of burnout) (Lasalvia *et al*., 2021). Not at least, the fear associated with the coronavirus pandemic and the consequent lockdown, had a relevant impact on psychological wellbeing (Naqvi, 2020).

The World Health Organization (WHO) declared that mental health care should be considered one essential health service to be maintained throughout the COVID-19 health crisis for different populations (World Health Organization, 2020). Nevertheless, a study conducted in China (Zhong *et al*., 2020) describes that 83% of participants with perceived mental health needs ascribed their lack of help-seeking to barriers to accessibility and availability of mental health services. In fact, the pandemic has also brought the need for fast and flexible adaptations in the health organisation to balance the increased demand (Kuzman *et al*., 2021) and the contact's reductions among patients and between patients and professionals (Pignon *et al*., 2020). The main challenges occurred in day and residential care: most day facilities have been temporarily closed, and residential patients have had limited permission or no leave (Carpiniello *et al*., 2020). Other changes have affected outpatient care, with national and regional rules limiting interventions to most urgent cases. Home visits, a common practice in most Community Mental Health Services (CMHSs), have been drastically reduced (de Girolamo *et al*., 2020) and telepsychiatry has been employed worldwide to deliver assessments and clinical follow-up for psychiatric outpatients (Gentile *et al*., 2022).

Despite this critical context, in our knowledge there is a lack of quantitative evidence that observe the longitudinal trend of access to mental health services in Italy and their consequences on mental health patients in a long period of time (Meloni *et al*., 2020). Some quantitative studies have reported variations both in the frequency of outpatient psychiatric interviews and in the composition of users, with a reported increase in requests; others reported a general description of the change in mental health services mainly during or shortly after the lockdown period. However, no study has explored changes in frequency and type of mental health access occurring during the first pandemic year in a large area served by an integrated community-based mental health system.

This study aims to fill this gap by evaluating the association between the severity of pandemic restriction in Italy and the mental health services’ utilisation and, specifically by looking at diagnoses and type of contacts most affected by lockdown and restrictions.

## Methods

### Study design

We conducted a retrospective analysis by using an administrative database. This study was designed to: (1) evaluate the association of the lockdown and the intermediate restrictions due to the COVID-19 pandemic and the mental healthcare provision by comparing the access to community mental health care between 2019 and 2020 in the Mental Health Department of the Verona catchment area; (2) to explore the effect of clinical characteristics (i.e., psychiatric diagnoses) and types of contacts on changes in mental health contacts occurring over the two years considered.

Lockdown refers to the time-period from 9 March 2020 to 3 May 2020 (8 weeks). Intermediate restrictions are part of the timeframe period from 9 May 2020 to 31 December 2020. This timeframe is not continuous but was identified based on an index (see the ‘Estimate of the effect of lockdown and intermediate restriction on the number of contacts’ section below for more details).

The research project complied with the principles of the Declaration of Helsinki regarding medical research in humans and it was approved by the local Ethics Committee (3327CESC, Prot. 35819, 14 June 2021).

### Setting

This study was conducted in the Verona catchment area, located in the Veneto Region (northeastern Italy). The main agency providing psychiatric care for the adult population is the Department of Mental Health, which covers a population of approximately 924 742 inhabitants, comprises five psychiatric acute inpatient wards located in five general hospitals. Outside the general hospital, a network of community mental health centres, each serving a well-defined catchment area, provides outpatient care and community care in close conjunction with other health and social local services. The Department of mental health also provides rehabilitation and recovery programs through day-centres and residential facilities, in-house and domiciliary services and collaborate with welfare, housing and educational services.

### Data sources

Since 1978, the year of implementation of the Italian psychiatric reform, a Psychiatric Case Register (PCR) routinely records, for all subjects seeking psychiatric and/or psychological care, sociodemographic characteristics, ICD-10 diagnoses, past psychiatric and medical history, clinical data, in-patient mental health care and outpatient contacts (Tansella and Burti, 2003). The PCR is a local database that includes routinely information from the Mental Health Department of Verona collected by mental health professionals; the resulting data become part of the Veneto Region Information System and then transmitted and included into the Italian Mental Health Information System. The PCR has been an extremely reliable and useful tool to monitor and evaluate the epidemiology of mental disorders and the provision of mental health care in this large catchment area (Amaddeo *et al*., 1997, 2001, 2007).

### Contacts and type of services

For each patient, mental health contacts were grouped into five components (supplementary material): (1) *outpatient care*, which includes all contacts at the outpatient and community level, mainly delivered by psychiatrists, clinical psychologists and community nurses; (2) *social and supportive interventions*, which includes provision of social services by mental health staff (especially social workers) as well as home visits, visits within healthcare facilities run by other agencies or visits to the premises of voluntary organisations; (3) *rehabilitation interventions*, which covered all contacts at rehabilitation groups and individual rehabilitation interventions delivered at the day centres mainly by occupational therapists and psychiatric rehabilitation therapists; (4) *multi-professional assessments*, which includes meetings of members of mental health staff with those of other healthcare or social agencies or among mental health staff; (5) *day care,* derived from the number of days spent at day centres, in a public sector specialized mental health care.

These five components represent the functional (but not organisational) components of the community mental health care and also provide a logical set of categories according to which to evaluate changes in the utilisation of mental health services during the COVID-19 pandemic.

### Data collection and statistical analyses

#### Study sample

The study included all contacts made by adult patients (>18 years old) with the services of Mental Health Department of the Verona catchment area between 1 January 2019 and 31 December 2020. All type of contacts (with psychiatrists, psychologists, nurses, psychiatric rehabilitation therapists, occupational therapists and social workers) provided by mental health services were included.

#### Comparison between 2019 and 2020

The number of mental health contacts occurring over each year was calculated, both globally and for each type of service. The number of patients with at least one contact in each year was also calculated, both globally and for each group of given sociodemographic, contextual and clinical variables.

As for sociodemographic variables, citizenship (‘Italian’, ‘other’), age bands (‘18–24’, ‘25–44’, ‘45–64’, ‘⩾65’), marital status (‘single’, ‘married’, ‘separated/divorced/widower’), living situation (‘alone’, ‘with family members’, ‘shelter/residential facility’) and gender were considered. Moreover, the number of patients with at least one contact in each year was computed both by degree of urbanisation at municipal level with data from EuroStat (Dijkstra and Poelman, 2014) and by diagnostic groups (ICD-10 codes, WHO, 1992) as follows: schizophrenia and related disorders (codes F20 to F29), affective disorders (codes F30 to F39); neurotic or somatoform disorders (codes F40 to F48) and other diagnoses (all other ICD-10 F and Z codes).

Both in the case of the number of contacts and of the number of people, equality of between year 2019 and 2020 was tested, in all cases, through the variance test for Poisson-distributed variables (Cochran, 1954).

#### Estimate of the effect of lockdown and intermediate restriction on the number of contacts

The dataset is made up by the weeks of the years 2019 and 2020 (by considering the length of the last week of 2019 8 days and of the last week of leap year 2020 9 days, so that no day of the year will be excluded). The main variable of interest was the level of restrictions related to the pandemic emergency. In particular, the ‘Covid Stringency Index’ (CSI) related to Italy (and measured daily) was used in order to divide the weeks of year 2020 (and of year 2019, by comparison) into three classes: ‘no or reduced restrictions’ (average CSI below 0.7), ‘intermediate restrictions’ (average CSI between 0.7 and 0.8) and ‘lockdown’ (average CSI above 0.8).

The ‘Covid Stringency Index’ is an indicator, ranged from 0 to 1, created by a group of researchers from the University of Oxford (Hale *et al*., 2021) to estimate the level of restrictions related to the containment measures of the pandemic in the various states of the world. For a given country, the CSI value was obtained by considering, for each day of the year, nine different variables, including any decisions regarding the limitation of individual travel or the closure of schools, economic and work activities.

A negative binomial regression model was chosen, to take into account both the discrete nature of the outcome and the presence of overdispersion. In order to take into account that not all weeks have the same number of working days (due both to public holidays and to the higher length of the last week of each year), such variable was inserted as an exposure (making the outcome ‘number of contacts per working day’). However, since, from one side, in non-working days there could still be contacts since the emergency service is guaranteed and, from the other side, there could be less contacts in weeks where public holidays are present, the fraction of working days in the week will be inserted as a control variable in the regression. Furthermore, since the COVID-19 restrictions include the travel ones, we will insert an indicator variable for weeks that included public holidays in days where inter-regional travels were either banned or discouraged.

A ‘difference in differences’ (Higgins *et al*., 2013) approach was used: in particular, the difference of the number of contacts between 2019 and 2020 on the weeks of lockdown and intermediate restrictions was compared with the same difference in weeks of no or reduced restrictions, and such difference was interpreted as the estimated effect of restrictions. To do so, the following indicators were inserted in the regression: one related to year 2020 and two related to the period of the year (one for the weeks that had intermediate restrictions in 2020 and one for the weeks that were lived in lockdown in 2020). The two interactions between the year-variable and the restriction-variables are our effects of interest.

Both a global regression on all contacts and separate regressions for each type of service were performed and Incidence Rate Ratios (IRRs) were calculated. A global test on equality of the two coefficients related to the effects of restrictions across service type was also performed, in order to assess whether there is evidence of systematic differences across service types. All analyses were performed using Stata 17 (StataCorp, 2021).

## Results

Table 1 compares personal characteristics of patients who had at least one contact with the community mental health system of the Verona catchment area in 2019 (*n* = 9592) and in 2020 (*n* = 8680). A significant reduction in number of patients who had mental health contacts was registered in 2020, both overall and in almost all the categories considered, except for people aged 18–24 years (−5.7%; *p*-value 0.290), for foreign-born population (−7.4%; *p*-value 0.090) and for patients with a diagnosis of schizophrenia and related disorders (−5.0%; *p*-value 0.122). The difference between the number of patients who had mental health contacts in 2019 and in 2020 was not statistically significant even for first-ever patients with a diagnosis of schizophrenia and related disorders.

**Table 1.** Comparison between numbers of patients who had access to mental health services in 2019 and 2020 by socio-demographic and diagnostic characteristics

	2019	2020	*X*^2^	Δ 2019–20 (%)	*p*-value
**All patients**	9592	8680	45.5	−912 (9.5%)	**<0**.**001**
Gender					
Female	5215	4693	27.5	−522 (10.0%)	**<0.001**
Male	4377	3987	18.2	−390 (8.9%)	**<0.001**
Age					
18–24 years	664	626	1.1	−38 (5.7%)	0.290
25–44 years	2463	2244	10.2	−219 (8.9%)	**0.001**
45–64 years	4388	3976	20.3	−412 (9.4%)	**<0.001**
≥65 years	2077	1834	15.1	−243 (11.7%)	**<0.001**
Citizenship	(1 missing)	(1 missing)			
Italian	8574	7737	43.0	−837 (9.8%)	**<0.001**
Others	1017	942	2.9	−75 (7.4%)	0.090
Marital status	(540 missing)	(584 missing)			
Single	4234	3911	12.8	−323 (7.6%)	**<0.001**
Married	3390	3000	23.8	−390 (11.5%)	**<0.001**
Separated/divorced/widower	1428	1185	22.6	−243 (17.0%)	**<0.001**
Living situation	(1127 missing)	(1026 missing)			
Alone	1370	1185	13.4	−185 (13.5%)	**<0.001**
With family members	6705	6170	22.2	−535 (8.0%)	**<0.001**
Sheltered or residential facility	390	299	12.0	−91 (23.3%)	**0.001**
Urbanicity level	(178 missing)	(148 missing)			
Urban	2677	2489	6.8	−188 (7.0%)	**0.009**
Intermediate	5098	4643	21.3	−455 (8.9%)	**<0.001**
Rural	1639	1400	18.8	−239 (14.6%)	**<0.001**
Diagnosis	(164 missing)	(146 missing)			
Schizophrenia and related dis.	1898	1804	2.4	−94 (5.0%)	0.122
Affective disorders	2268	2016	14.8	−252 (11.1%)	**<0.001**
Neuroses and somat. disorders	2758	2476	15.2	−282 (10.2%)	**<0.001**
Other diagnosis	2504	2238	14.9	−266 (10.6%)	**<0.001**
Diagnosis / First-ever patient	(29 missing)	(23 missing)			
Schizophrenia and related dis.	130	139	0.3	+9 (6.9%)	0.583
Affective disorders	545	405	20.6	−140 (25.7%)	**<0.001**
Neuroses and somat. disorders	1067	848	25.0	−219 (20.5%)	**<0.001**
Other diagnosis	926	703	30.5	−223 (24.1%)	**<0.001**

*Notes*. Significant results are highlighted in bold.

By considering the number of contacts, 57 096 (−33.9%) fewer mental health contacts in 2020 were registered with respect to 2019 (*p*-value < 0.001). Such difference remained statistically significant (*p*-value <0.001 in all cases) across the various type of contacts (Table 2), although with a different magnitude. Rehabilitation interventions and number of days spent at day-centres had the greatest decrease over 2020, showing respectively a reduction of 24 105 (−50.6%) and 20 132 (−63.4%) compared to 2019. Conversely, social and supportive interventions had the lowest reduction in 2020, with 1177 (−5.0%) fewer contacts than 2019. Multi-professional contacts and outpatient contacts showed an intermediate pattern trend between the previous categories, with respectively 555 (−37.9%) and 11 127 (−17.5%) fewer contacts in 2020 as compared with 2019.

**Table 2.** Comparison between numbers of MH contacts registered in 2019 and 2020 by type of contacts

Type of contact	2019	2020	X^2^	Δ 2019-20 (%)	*p-value*
Outpatient care	63 659	52 532	1065.6	−11 127 (17.5%)	**<0**.**001**
Rehabilitation intervention	47 642	23 537	8163.2	−24 105 (50.6%)	**<0.001**
Multi-professional assessment	1463	908	129.9	−555 (37.9%)	**<0.001**
Social and supportive interventions	23 738	22 561	29.9	−1177 (5.0%)	**<0.001**
Day care	31 776	11 644	9334.3	−20 132 (63.4%)	**<0.001**
Overall contacts	168 278	111 182	11 665.2	−57 096 (33.9%)	**<0.001**

*Notes*. Significant results are highlighted in bold.

Negative Binomial regressions (Table 3) displayed a statistically significant estimated effect of lockdown, but not of intermediate restrictions, in terms of reduction in number of contacts. In particularly, the overall reduction in number of contacts ascribable to the lockdown is 32.7% (IRR 0.673; *p*-value <0.001). All type of contacts showed a decreasing effect of the lockdown, except for social and supportive interventions (IRR 1.082).

**Table 3.** Estimated effect of lockdown and restrictions by type of contact

Type of contact	Lockdown		Intermediate restrictions	
	IRR	CI	IRR	CI
Outpatient care	**0**.**764**	**(0**.**639; 0.913)**	0.969	(0.813; 1.155)
Rehabilitation intervention	**0.517**	**(0.381; 0.700)**	0.854	(0.636; 1.146)
Multiprofessional assessment	**0.107**	**(0.047; 0.241)**	**0.406**	**(0.196; 0.841)**
Social and supportive interventions	1.082	(0.864; 1.356)	1.240	(0.995; 1.546)
Day care	**0.068**	**(0.024; 0.187)**	1.089	(0.462; 2.565)
Overall contacts	**0.673**	**(0.539; 0.840)**	0.987	(0.796; 1.224)

*Notes*. IRR, *Incidence Rate Ratio*; IC, Confidence Interval; SE, Standard Error.

Significant results are highlighted in bold.

Overall, a lack of statistical significance (*p*-value 0.905) and a very small associated point estimate (IRR 0.987, corresponding to a 1.3% reduction) was found for intermediate restrictions. However, they were estimated to more than halve the number of multi-professional assessments (−59.4%; *p*-value 0.015). On the contrary, despite a 24.0% increase, the effect on social and supportive interventions marginally failed to reach statistical significance (*p*-value 0.056); all the other types of contacts showed small and non-significant effects.

**Fig. 1.**
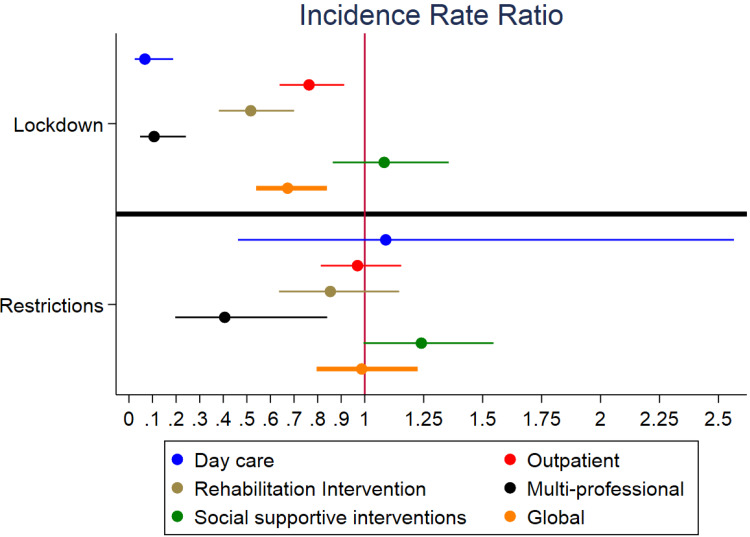
Estimated effect of lockdown and restrictions by type of contact *Notes*: *Incidence Rate Ratio* on X axis, levels of stringency on Y axis.

A graph of the results of negative binomial regressions is reported in Fig. 1.

## Discussion

To our knowledge, this is one of the first studies that analysed the effects of the COVID-19 pandemic on utilisation of community-based mental health services using routine collected data and a ‘difference in differences’ approach comparing 2019 and 2020 on the weeks of lockdown, intermediate restrictions and no restrictions. In contrast to findings reported by nation-wide surveys conducted over the same period (Fiorillo *et al*., 2020; Moccia *et al*., 2020; Vahratian *et al*., 2021) we did not find an increase in the number of patients making their first-ever contact for depression or anxiety disorders. Conversely, we found an overall reduction in the number of patients asking for mental health care in 2020 as compared to 2019, and this reduction applies to every diagnostic group considered. This was probably due to some limitations in the accessibility of healthcare services in general, due to national and regional legislations, together with the fear of being infected by the virus, that might have prevented many patients from attending healthcare facilities (Carpiniello *et al*., 2020). Moreover, the reduction in number of patients seeking mental health help may be related to organisational changes and restrictions that occurred in most Italian mental health facilities to reduce the spread of the virus (de Girolamo *et al*., 2020). It may be also possible that some people might have asked for mental health care to their GPs or to other private agencies with lower barriers to access.

When stratifying the population by sociodemographic and diagnostic characteristics, we found that the reduction in the number of patients asking for mental health care in 2020 remained statistically significant for every considered category, except for patients with schizophrenia and related disorders, for younger people (aged 18–24 years) and for the foreign-born population. Such fining might be interpreted as the result of a higher average clinical severity within those subgroups, which might have encouraged both patients and mental health professionals to maintain regular mental health contacts even in a period of restrictions due to the pandemic.

As for the foreign-born population it is known that first contact in emergency department and first contact as psychiatric inpatient occurred more often in migrants than their autochthonous counterpart (Cristofalo *et al*., 2018; Graetz *et al*., 2017). Such supposed higher average clinical severity in this subgroup, might have reduced for such patients the otherwise common tendency to avoid or delay usual care during 2020 because of the pandemic and its related restrictions. Nevertheless, this is a controversial interpretation: some authors underlined the possible presence of some additional barriers in the access to mental health services during pandemic, which might have specifically affected some subgroups of patients of foreign origin like, e.g., migrants and asylum-seekers (Aragona *et al*., 2020).

The lack of reduction in mental health contacts for young adults during 2020 was probably due to the fact that they represent a particularly vulnerable population that experienced particularly high levels of distress during pandemic as compared to other age groups (Talevi *et al*., 2020). That was probably related to some mental health risk factors, such as frequent unemployment condition, social isolation experienced during lockdown and high level of distress related to the concern about COVID-19 infected relatives or acquaintances (Liang *et al*., 2020; Moreno *et al*., 2020). Another possible reason might be the higher average familiarity of youths with technologies often involved in telepsychiatry (Nicholas *et al*., 2021), allowing for a more extensive use of this tool and thus reducing the number of young patients who might have faced a delay or an avoidance of the usual mental health services in 2020. It is important to underline that all the three subgroups not showing a reduction in mental health contacts over 2020 had a small sample size that may explain the lack of statistical significance in the small reductions recorded within them. Therefore, the findings discussed above need to be interpreted with caution and need to be confirmed on larger samples.

Finally, we found a lower contacts reduction in 2020 for patients living in urban areas (−8.8%) as compared to those living in rural areas (−14.5%). A possible explanation of this difference could be related to the fact that people living in rural areas are likely to experience worse mental health conditions than urban populations due to poorer access to mental health services and lower levels of psychological support (Gardiner *et al*., 2020; Moon and Moon, 2020).

When comparing the trends of different kinds of contacts in 2020, we found clear evidence that the most important decrease regarded rehabilitation interventions and the day-care activities, confirming what already emerged from the literature (Carpiniello *et al*., 2020). This is an expected finding as in Veneto regional legislation required that day-care centres should have been totally closed over the first six months following the pandemic onset. This decision was taken as activities within day-care centres are delivered in groups, with possible greater difficulties in maintaining the prescribed safety distance (D'Agostino *et al*., 2020). Furthermore, they include manual, artistic and expressive activities, that are difficult to be delivered through telemedicine (or telerehabilitation). On the contrary, some services (e.g., outpatient care or multi-professional assessments) may allow an easier respect of the interpersonal safety distances and they are easier to be delivered by online devices (Kuzman *et al*., 2021).

Moreover, our data showed that social and support interventions were the category with the lowest decline between the two years considered. This finding is consistent with the perception of psychiatric patients as more fragile than the general population during pandemic, because of the common presence of previous social, economic and work difficulties (Diaz *et al*., 2021). Then, this was a plausible effort, made by mental health services all over the country, to support this vulnerable population, through an increase in social and support interventions. Finally, the provision of some services has undergone a reorganisation, going to change the definition and subsequent classification in the PCR (e.g., in 2019 the personal delivery of psychotropic medication to the more difficult to treat patients was mainly carried out within mental health care facilities and thus recorded as ‘outpatient care’, whereas in 2020 this kind of service was frequently provided at patients’ home and thus registered as ‘social and support intervention’).

By using regression analysis, we found that most of the reduction observed in the number of contacts in 2020 was attributable to the lockdown period; in other words, the lockdown led to an estimated effect of reduction in the global number of contacts with the CMHSs considered of about one-third. That means that the hardest period of pandemic has therefore severely limited the access to mental health care for patients in need. The only exception to this trend was social and supportive interventions: they showed an estimated effect of increase, both during lockdown and intermediate restrictions periods. One possible reason for this finding might be that, as the pandemic progressed, there might have been an increase in social and administrative issues in the population, thus increasing the number of patients who looked for help from CMHSs in managing them in the second part of 2020 (period of intermediate restrictions). Another possible consideration is that, during the period of intermediate restrictions, there might have been fewer barriers in the access to services, thanks to a greater availability of personal protective equipment and to a prompt reorganisation of the services (Fagiolini *et al*., 2020). That may explain both the stronger estimated effect of increase attributed to intermediate restrictions on social and supportive interventions (e.g., allowing to gradually restart interventions like home visits), and the lack of statistical significance of their estimated effect on the other types of contacts.

Caution is required in interpreting our findings, and some important methodological limitations, should be acknowledged. First, our study could be affected by information bias because the dataset was collected by different teams of psychiatrists. In fact, we found some missing data. Second, due to the structure of the dataset, it was not possible to isolate the variable ‘telematics contacts’, which would have given significant contributions to the interpretation of the data. Due to the nature of the data collected, important clinical information regarding the severity of the cases considered and the combined effects of the pandemic on the other health care services are also lacking, again limiting our possibility to draw conclusions from the data. Third, we were unable to explore the clinical characteristics of children and adolescents (<18 years) on changes in mental health contacts occurring over the two years considered. Finally, our study was conducted in a single Mental Health Department, which limits the generalisability of our results to other contexts. Despite these limitations commonly affected the broad field of retrospective epidemiological studies, future studies should consider these lacking to provide more relevant clinical evidence.

Despite these limitations, our study has important implications. Our findings describe the trend of mental health utilisation during the first year of the COVID-19 pandemic, thanks to an extremely reliable routine dataset. Despite access to community mental health care during a pandemic remains problematic, the community mental health system in the Verona catchment area has shown to be able to support more vulnerable and severely ill patients, by maintaining continuity of contact and day-by-day support through social and supportive interventions. One significant aspect is that we used an index (CSI) that is available for most countries around the world (Hale *et al*., 2021). This could facilitate similar studies in other countries and their comparison with our local data. Moreover, a complex statistical methodology was applied: the difference-in-differences allowed to reduce confounding factors and thus to estimate the effect of pandemic restrictions more precisely. Finally, there is a need for more studies on this topic, by e.g. correlating changes in access to CMHSs with those in mental health emergency access, which could better reflect the burden of COVID-19 pandemic both on general population and on psychiatric patients. Future studies could also provide information on the long-term effects of COVID-19 pandemic on mental health and well-being, and on its consequent increase of mental health services utilisation and changes in diagnostic casemix.

## Data Availability

Data of the present study will be made available upon motivated request.

## Supplementary material

For supplementary material accompanying this paper visit http://doi.org/10.1017/S2045796023000100. click here to view supplementary material
