# Supplementary material for: Effect of COVID-19 pandemic on utilisation of community-based mental health care in North-East of Italy: A psychiatric case register study
Source: Epidemiol Psychiatr Sci. 2023 Apr 11;32:e17. doi: 10.1017/S2045796023000100 (PMC10130733; doi:10.1017/S2045796023000100)
Supplement: Supplementary file 1 [file S2045796023000100sup001.docx]

**Effect of COVID-19 pandemic on utilization of community-based mental health care in North-East of Italy. A psychiatric case register study**

**Prina and collaborators**

Supplementary Material

**Table S1: Mental health contacts evaluated in the study.**

| Component | Description |
| --- | --- |
| Outpatient care | - First psychiatric visit - First psychological visits - Specialist check-up visit - Psychiatric interview - Clinical psychological interview - Clinical report - Specialist consultation-liaison - Individual psychotherapy - Couple psychotherapy - Family psychotherapy - Group psychotherapy - Assessment through standardised tools - Psychiatric somatotherapy - Psychoeducation (individual family) - Psychoeducation (multiple families) |
| Social and supportive interventions | - Home visits - Supportive intervention - Family interview - Social support intervention - Intervention for administrative matters |
| Rehabilitation interventions | - Basic-skills intervention (individual) - Basic-skills intervention (group) - Expressive and/or physical intervention (individual) - Expressive and/or physical intervention (group) - Job training - Re-socialization intervention (individual) - Re-socialization intervention (group) - Stays |
| Multi-professional assessments | - Operational team-meeting - Meeting between professionals |
| Day care | - Day spent in a day centers (less than 4 hours) - Day spent in a day centers (more than 4 hours) |
